# Supplementary material for: Intestinal Goblet Cell Loss during Chorioamnionitis in Fetal Lambs: Mechanistic Insights and Postnatal Implications
Source: Int J Mol Sci. 2021 Feb 16;22(4):1946. doi: 10.3390/ijms22041946 (PMC7920290; doi:10.3390/ijms22041946)
Supplement: Supplementary file 1 [file ijms-22-01946-s001.pdf]

SUPPLEMENTARY FIGURES

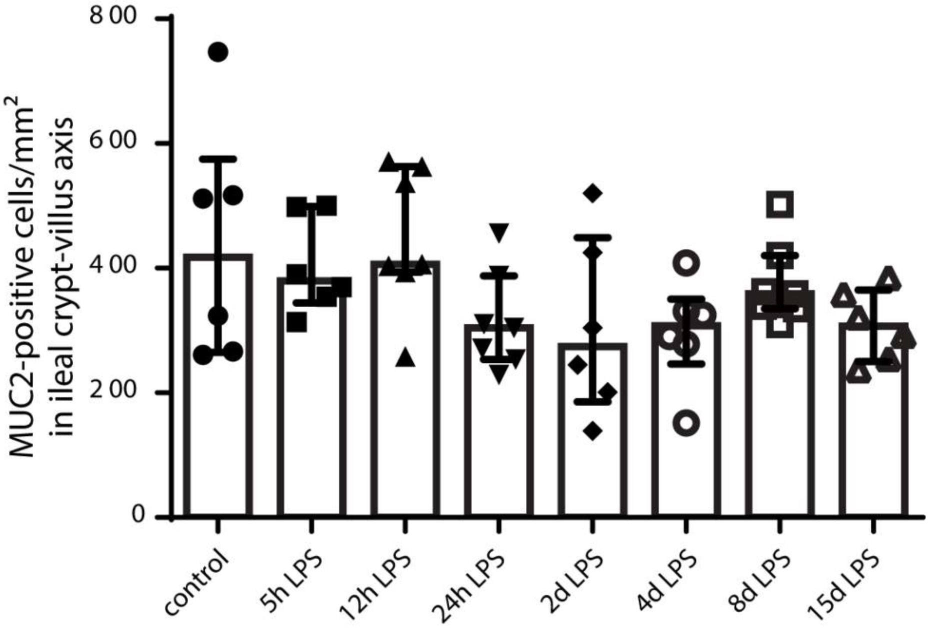

Supplementary figure 1. Total MUC2+ cell count in intestinal crypt-villus axis. No statistically significant changes were observed between the different treatment groups. Each data point represents the MUC2-positive cell count of one lamb. Data are displayed as median with interquartile range.

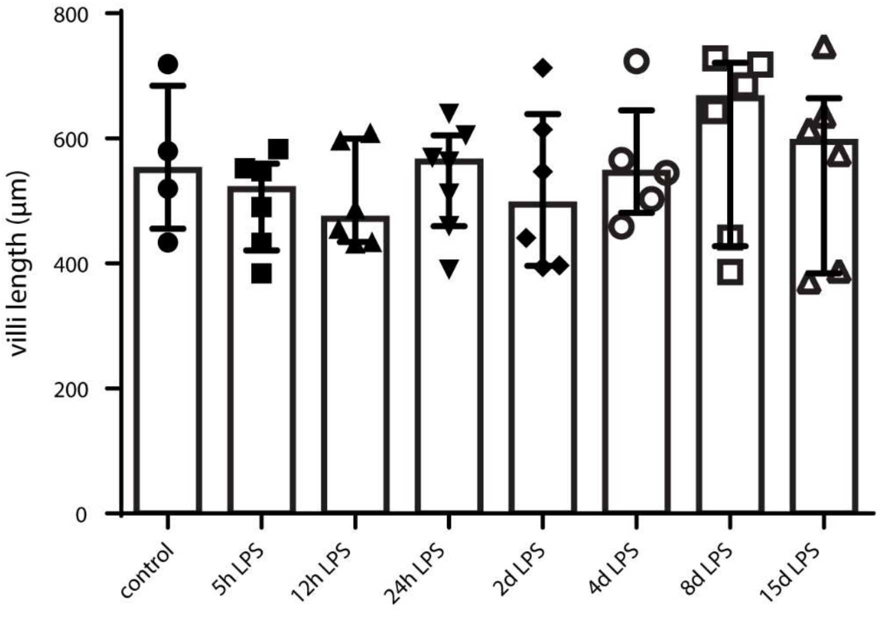

Supplementary figure 2. Villus length measurements of all experimental groups. No statistically significant changes were observed between the different treatment groups. Each data point represents the villus length of one lamb. Data are displayed as median with interquartile range.

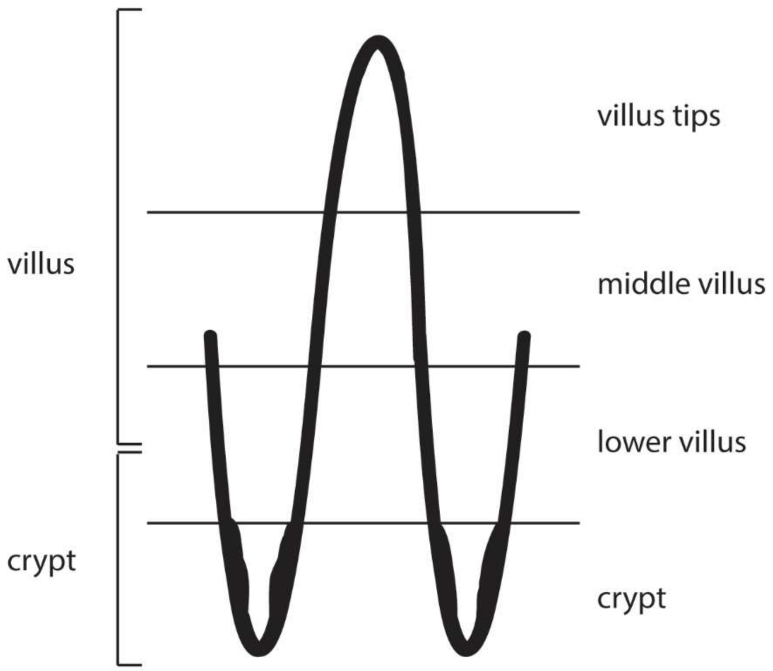

Supplementary figure 3. Division of crypt-villus axis in crypt, lower villus, middle villus and villus tips.
